# Research on the deformation mechanisms of accumulated landslides induced by different rain patterns based on flume model tests

**DOI:** 10.1371/journal.pone.0329728

**Published:** 2025-08-28

**Authors:** Wu Yi, Hong Luo, Xiaohu Huang, Xiaohan Zhu, Zhengyu Wang, Yong Li

**Affiliations:** 1 College of Civil Engineering and Architecture, China Three Gorges University, Yichang, China; 2 National Field Scientific Observation and Research Station for Landslides in the Three Gorges Area, Yichang, Hubei, China; 3 Xingshan County Land Consolidation Center, Yichang, Hubei, China; 4 Hydrogeological and Engineering Geological Brigade, Hubei Provincial Bureau of Geology, Jingzhou, Hubei, China; Auckland University of Technology, NEW ZEALAND

## Abstract

In the context of intensifying global environmental pressures, heavy rainfall in extreme climate regions significantly increases landslide risks, threatening societal stability and sustainable development. While research on rainfall-induced landslides is well-established, the deformation and instability mechanisms of landslides under complex rainfall patterns warrant further investigation. This study focuses on the Wangjiapo landslide in the Three Gorges Reservoir area. Through comprehensive field investigations, deformation monitoring, and rainfall data analysis, we systematically characterized the landslide’s deformation characteristics. Employing the similarity theory, a flume model experiment was designed to simulate four distinct rainfall patterns. Real-time monitoring of parameters, including slope displacement, pore water pressure, soil pressure, and moisture content, was conducted using multiple sensors, such as pull wire displacement sensors, pore water pressure sensors, and soil pressure sensors. The macroscopic deformation and internal stress variations of the landslide under varying rainfall conditions were thoroughly analyzed. Statistical processing of experimental data facilitated a comparative analysis with in-situ monitoring data, with further validation performed using Geo-Studio numerical simulation methods. Through these integrated approaches, this study elucidates the influence of different rain patterns on the deformation and failure mechanisms of accumulated landslides. Our findings highlight the critical role of rainfall intensity and rainfall time series in driving landslide deformation, identifying pore water pressure and shear strength variations as crucial factors inducing landslide instability. Furthermore, we delineate four distinct stages of the landslide failure process and characterize the temporal and spatial evolution of the instability mechanism, addressing a critical gap in understanding the deformation mechanisms of landslides under complex rainfall patterns. These results provide valuable insights for landslide monitoring and early warning systems and inform strategies for landslide disaster monitoring and prevention.

## Introduction

Landslides rank among the most devastating natural hazards, surpassed only by earthquakes in destructive potential. They result in significant loss of life and substantial economic damages globally [1–5]. Statistical analyses indicate that landslides account for approximately 17% of fatalities attributed to natural disasters [6]. In China, where mountainous and hilly regions constitute two thirds of the land area and exhibit complex geological and diverse climatic conditions, landslides are both frequent and widespread [7–9]. Between 2000 and 2015, China recorded 373,630 landslide events, causing 10,996 fatalities and annual economic losses amounting to billions of dollars [10,11]. Beyond economic and human tolls, landslides pose severe threats to the safe operation of critical infrastructure, such as highways [12]. Consequently, comprehensive investigations into the triggering factors and induced mechanisms of landslides [13–15] are crucial for developing effective prevention and mitigation strategies. In response to this pressing challenge, the China Disaster Reduction Committee [16] has set a goal to modernize disaster prevention and mitigation capabilities by 2035, aiming to enhance the efficiency, coordination, and effectiveness of responses to major natural hazards.

With the intensification of global climate change, extreme weather events, particularly heavy rainfall, have become increasingly frequent, exacerbating the occurrence of landslides. Recent advancements in landslide research have revealed the complex and multifaceted nature of their formation mechanisms. Among the numerous natural and anthropogenic factors influencing landslide stability, heavy rainfall is recognized as a crucial factor in triggering landslide events [17–20], with a significant role in the initiation of debris flow landslides. Statistical analyses from Zigui County, situated in the Three Gorges Reservoir area along the Yangtze River, indicate that of 154 recorded debris flow landslides with volumes exceeding 500,000 cubic meters, 149 were induced by rainfall, accounting for 96.75% [13]. As a recurrent geological hazard, rainfall-induced landslides cause substantial economic losses and casualties [21,22]. Consequently, elucidating the induced mechanisms of rainfall-induced landslides and developing comprehensive landslide monitoring and early warning systems are crucial for regional disaster prevention, mitigation, and sustainable development [23].

Investigating the deformation mechanisms of rainfall-induced accumulated landslides poses significant challenges in establishing comprehensive and reliable systems for landslide monitoring, early warning, and prevention. Recent research has made substantial contributions to understanding the deformation and failure mechanisms of accumulated landslides under rainfall influence, which can be categorized into three primary approaches.The first approach focuses on seepage and runoff generation based on saturated-unsaturated soil theory. Buscarnera et al. [24] examined how saturation events alter soil hydraulic properties, triggering flow landslides or liquefaction, thus providing novel insights into saturated-unsaturated soil dynamics. Liao et al. [25] analyzed the stability of high, steep loess slopes under varying hydrological conditions, integrating saturated and unsaturated soil properties to propose an enhanced stability analysis framework. Liu et al. [26] demonstrated that rainfall forms a seepage field within slopes, with seepage pressure acting on the front edge of the sliding mass, inducing slope deformation and failure. Wang et al. [27] investigated the progressive deformation mechanism of rainfall-induced accumulated landslides, revealing that rainfall infiltration through preferential seepage channels increases pore water pressure within tensile cracks, leading to soil softening and further crack propagation, which drives progressive collapse [28–30].The second approach employs mathematical models or numerical simulation methods to analyze rainfall infiltration and its impact on landslide stability. Liu et al. [31] utilized numerical simulations based on unsaturated seepage theory to show that rainfall infiltration enhances shallow rock mass saturation, reducing matrix suction in unsaturated zones. Li et al. [32] integrated field investigations with numerical modeling to develop a two-dimensional numerical model of a typical slope profile. Their study elucidated the mechanical mechanisms by which front edge erosion induces landslide deformation and quantified the response of landslide deformation to various influencing factors. Jia et al.[33] designed a centrifugal system to simulate water level fluctuations and employed numerical simulation methods to investigate the deformation characteristics of accumulated landslides. Their findings revealed the mechanisms underlying the progressive stabilization of the trailing edge of the landslide. Bordoni et al. [34] proposed a slope safety factor model based on volume moisture content, accounting for hydrological lag effects and pore water pressure changes, which effectively predicts shallow landslides. However, due to the complexity of the geotechnical slope structure, the relevant theory based on numerical simulation methods needs to be further developed to reveal the deformation mechanism of landslides. The third type is physical model experiments and centrifugal model tests. These methods are effective in studying the deformation characteristics and mechanisms of landslides.Zhou et al. [35] investigated the failure patterns of seepage-induced rock-soil mixed slopes through model tests, demonstrating that rainfall intensity and average slope angle significantly exacerbate slope instability. Hojat et al. [36] performed rainfall-triggered landslide model tests, identifying critical soil saturation, rainfall intensity, and rainfall duration as crucial factors influencing slope stability [37,38]. Zhang et al.[39] performed centrifugal model tests to evaluate the combined effects of reservoir water level fluctuations and rainfall, analyzing the deformation characteristics, pore water pressure, and soil pressure of landslides under these conditions. Wang et al. [40] utilized centrifugal tests to simulate the progressive failure of the Zhaojiagou landslide under prolonged low-intensity rainfall and groundwater seepage, revealing that low-intensity rainfall reduces effective stress and slope stability, triggering landslides.Despite significant advancements in theoretical models, numerical simulations, and experimental validations, these studies have greatly enhanced our understanding of the induced mechanisms, stability analysis, and landslide monitoring and early warning systems for rainfall-induced landslides. Nevertheless, the complex and dynamic nature of natural environments continues to pose challenges in fully explaining the instability mechanisms of accumulated landslides [13].

In the study of rainfall-induced landslides, the influence of rainfall intensity distribution on landslide failure modes has garnered significant attention from researchers. Fan et al. [41] developed a novel hydrodynamic triggering model to investigate the effects of asymmetrical rainfall intensity distribution, revealing that the peak intensity of initial rainfall exerts a pronounced influence on landslide failure. Ghani et al. [42] employed the limit equilibrium method and SLOPE/W software to assess landslide stability under varying rainfall patterns and intensities, demonstrating that initial rainfall predominantly governs the rate of decline in the slope safety factor. Zhao et al. [43] categorized Rainfall sequence into four distinct rainfall patterns and established a landslide triggering threshold model, finding that Rainfall time series significantly affect the triggering threshold. Specifically, when total rainfall remains constant, a pattern characterized by a weak initial phase followed by a strong phase is more likely to induce landslides. Zhang et al. [44] conducted flume model experiments to investigate the influence of dry density on rainfall infiltration in loess. Their findings revealed that, with increasing dry density, the infiltration lines under uniform rainfall, heavy rainfall, and torrential rain conditions exhibited distinct “Y,” “D,” and “∧” patterns, respectively. Despite extensive experimental analyses of landslide failure processes under different rainfall patterns, most studies have focused primarily on the role of initial rainfall, often overlooking the comprehensive failure process of landslides. Consequently, there is an urgent need to conduct in-depth investigations into the deformation characteristics and failure mechanisms of landslides throughout the entire rainfall duration to address this critical research gap.

Current research on the deformation mechanisms of rainfall-induced accumulated landslides [13,45–48] predominantly focuses on heavy rainfall or continuous rainfall events, with limited systematic exploration of instability patterns under complex rainfall patterns in flume model experiments. In natural settings, rainfall patterns exhibit significant variability, encompassing diverse intensities, durations, and distribution profiles, yet their impact on the instability of accumulated landslides remains underexplored. Future studies should prioritize the evolution of landslide deformation characteristics under varying rainfall patterns to enhance the precision of landslide monitoring and early warning systems and strengthen disaster prevention and mitigation capabilities. Addressing this research gap, the present study investigates the Wangjiapo landslide in the Three Gorges Reservoir area as a study case, employing a comprehensive analytical approach that integrates field investigations, long-term deformation monitoring, and rainfall data analysis to elucidate the deformation characteristics of landslides. This multifaceted approach provides a robust scientific foundation for understanding the induced mechanisms of accumulated landslide instability under rainfall influence. In this study, a flume model experiment was conducted to simulate the failure process of accumulated landslides under four distinct rain patterns, enabling an in-depth analysis of macroscopic deformation and internal stress variations. Experimental data were systematically compared with in-situ monitoring data to validate the findings. Additionally, Geo-Studio numerical simulation methods were employed to further corroborate the landslide deformation mechanism and the results of the physical model tests. Through these integrated approaches, we elucidated the hydromechanical evolution process of landslides, highlighting the critical influence of rainfall intensity and rainfall time series on landslide deformation. The study delineates the spatiotemporal characteristics of the instability mechanism, enhancing the understanding of the mechanisms driving accumulated landslide instability under complex rainfall patterns. These findings provide a robust scientific foundation for landslide monitoring and early warning systems and inform strategies for landslide disaster monitoring and prevention [49]. Moreover, the results contribute to the coordinated development of regional disaster prevention and mitigation efforts, supporting sustainable development. The study also offers significant practical value for landslide early warning and risk management [18].

## Study area and landslide data

### Study area

Xingshan County, situated in the northwest of Yichang City, Hubei Province, spans a total area of approximately 2,327 ${\text{km}}^{2}$. Its mountain ranges exhibit an east-west orientation, with geographic and geomorphic conditions characterized by elevated terrain in the east, northwest, and west, and lower elevations in the south, resulting in a maximum vertical elevation difference of 2,317.4 m. Located within the Three Gorges Reservoir area, the county is the birthplace of the historical figure Wang Zhaojun. In terms of geological structure, the study area forms part of the Qinling-Daba Mountain geological system, with mountainous regions comprising over 90% of the total area, predominantly exposing Ordovician and Cambrian carbonate rocks that are extensively distributed and relatively uniform in lithology. Climatically, the region is defined by a typical subtropical monsoon climate with abundant rainfall, exhibiting pleasant temperatures in spring and autumn, mild winters with brief low-temperature periods in river valleys, a trend toward warming and humidification in high-altitude zones, relatively balanced rainfall across seasons with a concentration in summer, synchronized precipitation and heat, and pronounced three-dimensional climatic characteristics.

**Fig 1:**
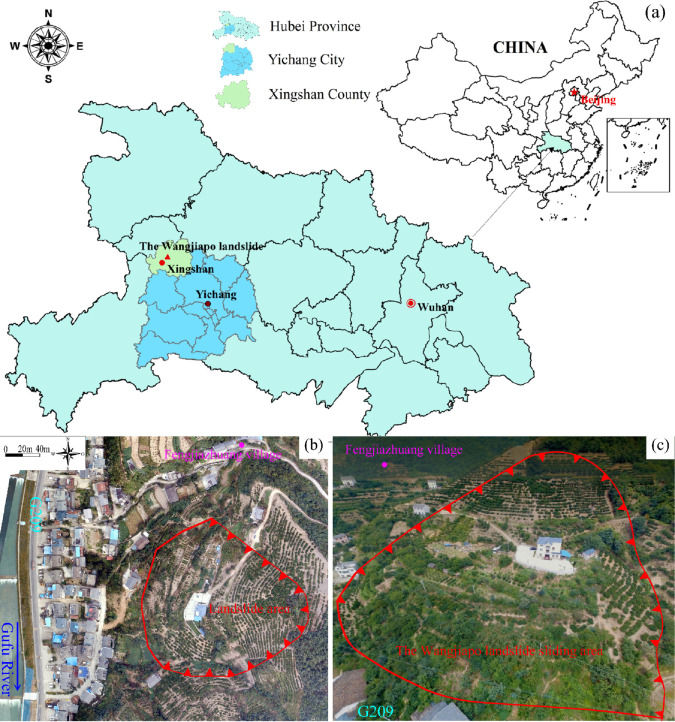
Location and geological map of the study area: (a) location of the study area; (b) satellite image of the slope; (c) overall view of the slope.

The rock mass in the study area (Fig 1) exhibits significant fragmentation due to intense faulting and jointing, resulting in complex geological conditions. In addition to the geological structure characterized by a lamellar cataclastic rock mass, the hydrogeological conditions and rainfall characteristics of the study area significantly exacerbate the risk of seepage-induced landslides.The regional hydrological system is primarily governed by the Gufu River, a tributary of the Yangtze River, characterized by steep topography and concentrated rainfall. In recent years, periodic fluctuations in water storage and discharge within the Three Gorges Reservoir area, coupled with the impacts of anthropogenic engineering activities, have exerted dual pressures on the geological environment, increasing the frequency of geological hazards. The combined effects of heavy rainfall, engineering activities, and reservoir water level fluctuations have led to the identification of 515 geological hazard sites across Xingshan County, with a cumulative volume of approximately 230million ${\text{m}}^{3}$. These hazards threaten the lives and property of 5,393 households, affecting 24,254 individuals, and have resulted in economic losses amounting to 220 million yuan.The complex topography and geological factors of the study area create favorable conditions for the occurrence of seepage-induced landslides. The distribution characteristics of landslide data are analyzed in detail below.

### Engineering geological properties

**Fig 2:**
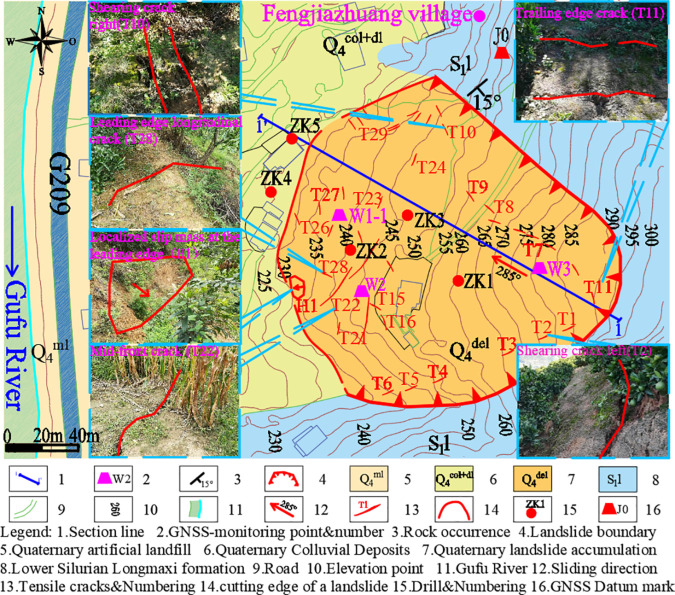
Engineering geological planar graph of the Wangjiapo landslide.

The Wangjiapo landslide is situated on the left bank of the Gufu River in Xingshan County, within a low mountain valley slope formed by structural erosion. The topography exhibits a northeast-high to southwest-low gradient, with an average slope angle ranging from 20^°^ to 30^°^ and an elevation span of 226 m to 296 m, resulting in a vertical height difference of 70 m. The landslide presents a broad, tongue-shaped morphology in plan view, with a north-south width of approximately 170 m and an east-west length of approximately 190 m. Its primary sliding direction is 285^°^, covering an area of approximately $3.23\;\times \;10^{4}m^{2}$, with an average thickness of 12 m and a total volume of approximately $3.88\;\times \;10^{5}m^{3}$, classifying it as a medium-sized accumulated landslide (Fig 2). Regionally, the landslide is located near the core of the Huangling Anticline within the Yangtze Craton, where the geological structure is relatively stable, with no significant faults or folds observed.

**Fig 3:**
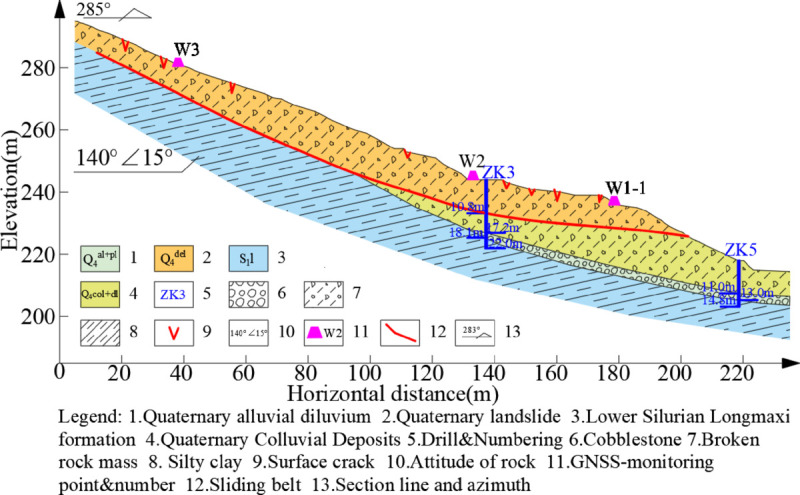
Engineering geological cross-section of the Wangjiapo landslide(1-1’).

Borehole data reveal that the sliding mass of the Wangjiapo landslide comprises silty clay with rubble, characterized by a soil-to-rock ratio of approximately 3:6. The rubble primarily consists of mudstone shale with angular to subangular edges, while the soil component is predominantly hard, plastic silty clay. The sliding belt, located at the deposit-bedding interface, consists of rubble and powdery clay, with a thickness of approximately 40 cm. This layer exhibits high viscosity and plasticity, allowing mud strips to be readily formed by hand. and the sliding bed consists of thin-layered mudstone shale from the Lower Silurian Longmaxi Formation (*S*_1_*l*), with a dip angle of the rock formation at 140^°^∠ 15^°^. The slope structure exhibits reverse slope characteristics, as illustrated in the typical structural profile (Fig 3). The study area is characterized by a subtropical monsoon climate, with rainfall concentrated from June to September, and the hydrological system is dominated by the Gufu River, a tributary of the Yangtze River. Groundwater in the study area comprises bedrock fracture water and pore water within loose deposits. Human activities, including building construction and road slope excavation, have significantly compromised the stability of slopes in the region.

### Monitoring systems and analysis

To monitor the deformation characteristics of the Wangjiapo landslide, an emergency monitoring plan was implemented, utilizing real-time automated GNSS surface displacement monitoring as the primary method, complemented by field geological investigations and rainfall monitoring data from the adjacent Jiangyangping landslide. The deformation mechanism of the Wangjiapo landslide was elucidated by analyzing the relationships between in-situ monitoring data and rainfall time series. The configuration of the monitoring system is illustrated in Fig 2-3.

**Fig 4:**
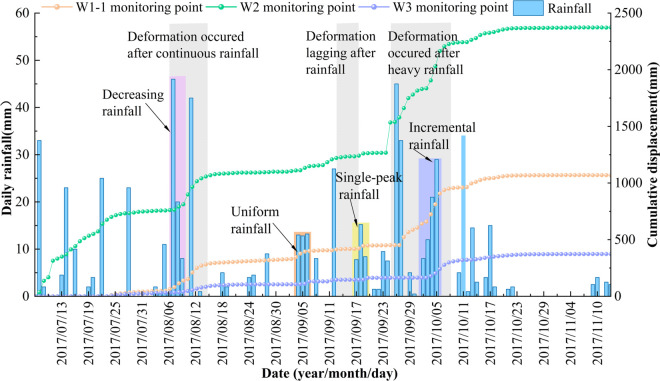
The cumulative displacement-rainfall-time relationships for Wangjiapo landslide.

The automated GNSS surface displacement monitoring system comprises three GNSS monitoring points (W0, W1-1, and W2-W3). From July 8 to November 13, 2017, a total of 129 in-situ monitoring data sets were collected. These data, combined with rainfall records from the adjacent Jiangyangping landslide in 2017, were used to generate a cumulative displacement-rainfall-time series relationship diagram (Fig 4). Monitoring results indicate that, since the implementation of professional monitoring, the Wangjiapo landslide has exhibited slow-moving deformation. Influenced by rainfall, the cumulative displacement curves at each monitoring point display multiple irregular step-like displacement patterns, suggesting a strong correlation between landslide deformation and rainfall time series. Consequently, the Wangjiapo landslide is classified as a typical seepage-induced landslide, with greater cumulative slope displacement at the front edge compared to the trailing edge, characteristic of a retrogressive landslide.

Based on the in-situ monitoring data, four distinct rain patterns were identified for the Wangjiapo landslide. From August 7 to 9, 2017, under decreasing rainfall conditions characterized by progressively diminishing daily rainfall, rapid infiltration at the front edge triggered significant increases in the cumulative displacement curves at monitoring points W1-1 and W2. As infiltration slowed, the cumulative displacement curve at W3, located at the trailing edge, exhibited a gradual rise. From September 4 to 6, 2017, uniform rainfall with relatively constant daily precipitation led to the progressive expansion of tension cracks from the front edge into the slope, resulting in steady increases in the cumulative displacement curves at W1-1 and W2. During the later stages, minor tension cracks developed at the trailing edge, with the cumulative displacement curve at W3 showing a gradual increase. From September 17 to 19, 2017, single-peak rainfall, marked by an initial increase followed by a decrease in daily precipitation, caused initial expansion of tension cracks at the front edge, with corresponding rises in the cumulative displacement curves at W1-1 and W2. Mid-rainfall, these cracks further expanded, sustaining the upward trend in W1-1 and W2 displacements, while tension cracks at the trailing edge began to extend, leading to a gradual rise in the W3 curve. From October 2 to 5, 2017, incremental rainfall with steadily increasing daily precipitation resulted in initial crack expansion at the front edge, with slow increases in the cumulative displacement curves at W1-1 and W2. As rainfall intensified, cracks at the central edge and front edge interconnected, causing significant rises in the W1-1 and W2 curves, while cracks at the trailing edge continued to expand, with the W3 curve showing a gradual increase. In the late stage of incremental rainfall, accelerated deformation was observed across the landslide, with all monitoring points exhibiting sustained increases in cumulative displacement.

### Deformation characteristics of the landslide

As of October 2017, the macroscopic deformation of the Wangjiapo landslide exhibited the following characteristics: the left and trailing edge boundaries were fully interconnected, with the right boundary showing progressive interconnection. Significant development of tension cracks was observed in the ground beneath structures at the central edge and in farmland at the front edge, with a relatively rapid deformation velocity. The shear outlet at the front edge was clearly defined, with muddy water continuously discharging post-rainfall, indicating the formation of a cohesive crack system approaching landslide instability.

## Experimental methodology

### Flume model design

**Fig 5:**
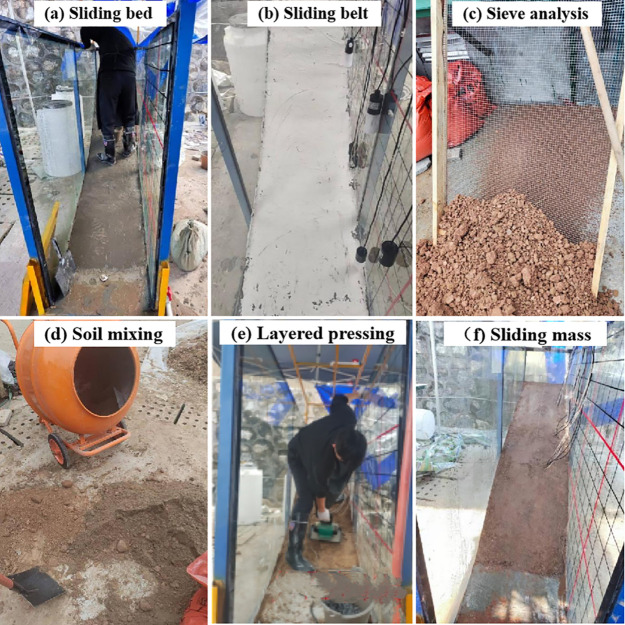
Flume model test production process.

To elucidate the deformation mechanism of accumulated landslides under varying rain patterns, this study compiled and statistically analyzed data on accumulated landslides in the Three Gorges Reservoir area, revealing distinct differences in deformation and failure mechanisms across different rainfall patterns. The Wangjiapo landslide, characterized by moderate thickness and slope angle, exhibits prominent tension cracks at the front edge and trailing edge, indicative of significant activity. Under the influence of rainfall, its deformation characteristics and failure process are particularly pronounced. Based on the landslide’s deformation history, material composition, and slope morphology, a representative geological section was selected for a flume model experiment, temporarily excluding the effects of boundary morphology and micro-topographic features. The model was stratified from top to bottom into a sliding mass, sliding belt, and sliding bed, reflecting the landslide’s geological structure. The geometric similarity ratio for the flume model was determined by integrating the geometric dimensions of the Wangjiapo landslide prototype with the flume model specifications. The flume model’s sliding bed has a longitudinal length of 2.7 m, a height difference of 1 m between the front edge and trailing edge, and an average slope thickness of approximately 0.17 m (Fig 5). In contrast, the prototype landslide features a longitudinal length of 190 m, a height difference of 70 m, and an average thickness of 12 m. According to similarity theory [50], the geometric similarity ratio (*C*_*l*_) is calculated as the ratio of prototype parameters (*L*_*p*_) to model parameters (*L*_*m*_), yielding $C_{l}=\frac{L_{p}}{L_{m}}\approx \frac{90}{2.7}\approx 70\text{ and }\frac{12}{0.17}\approx 70.$

### Similar materials

The flume model experiment is grounded in the principle of similarity, which allows for the simplification of complex landslide prototypes into scaled models to yield reliable experimental outcomes [51]. Prior studies indicate that, depending on research objectives, critical factors influencing landslide behavior can be prioritized, while secondary factors are excluded to streamline the model design. To ensure that the flume model experiment accurately reflects the deformation characteristics of the Wangjiapo landslide, the experimental design maintained close similarity in geometric dimensions and key physical properties between the model and the prototype, optimizing the fidelity of the results.

#### Geometric similarity

Based on the geometric similarity ratio and volumetric density similarity ratio [52], dimensional analysis and three fundamental similarity principles—geometric, kinematic, and dynamic—were employed to establish the similarity relationships for other relevant experimental parameters [53], as presented in Table 1. The primary parameters of the similar materials used in the experiment include the geometric similarity ratio (*C*_*l*_), density ($C_{\rho }$), gravitational acceleration (*C*_*g*_), internal friction angle ($C_{\varphi }$), Poisson’s ratio ($C_{\mu }$), displacement ($C_{\delta }$), cohesion (*C*_*c*_), deformation modulus (*C*_*e*_), permeability coefficient (*C*_*k*_), rainfall intensity (*C*_*q*_), and rainfall duration (*C*_*d*_). Utilizing dimensional analysis and the homogeneity theorem [54], these parameters are expressed in a dimensionless functional relationship as follows: f(l,ρ,g,φ,μ,δ,c,e,k,q,d)=0.

**Table 1:** Similarity coefficient table for the flume model test.

Physical Quantity	Similarity Constant Code	Similarity Coefficient
Geometric dimensions, *l*	*C*_*l*_	1: 70
Density, *ρ*	$C_{\rho }$	1: 1
Gravitational acceleration, *g*	*C*_*g*_	1: 1
Internal friction angle, φ	$C_{\varphi }$	1: 1
Poisson’s ratio, *μ*	$C_{\mu }$	1: 1
Displacement, *δ*	$C_{\delta }$	1: 70
Cohesion, *c*	*C*_*c*_	1: 70
Deformation modulus, *e*	*C*_*e*_	1: 70
Permeability coefficient, *k*	*C*_*k*_	1: 70^1/2^
Rainfall intensity, *q*	*C*_*q*_	1: 70^1/2^
Rainfall duration, *d*	*C*_*d*_	1: 70^1/2^

#### Similar physical properties.

The structural characteristics of the landslide prototype are detailed in the section describing the study area. Given the low moisture content of the shallow surface layer of the sliding mass and the high similarity in soil properties between the broken rock mass and silty clay, both are collectively referred to as the sliding mass in the simulation. Ensuring similarity in the mechanical parameters and soil properties of the sliding mass is crucial. Consequently, the saturated permeability of the sliding layer was determined through engineering geological analogy, involving a comprehensive analysis of lithology, particle size distribution, and moisture content. By integrating insights from previous research on landslide seepage and the sensitivity of similar material ratios [55,56], alongside references to landslide prototypes, flume model experiments, and tests of the physical, mechanical, and hydraulic properties of analogous materials, the mechanical parameters of the sliding mass and sliding belt were aligned closely with in-situ conditions. This process informed the final formulation of similar materials for the flume model, as presented in Table 2.

**Table 2:** Comparison of some parameters of prototype landslide and model landslide.

Landslide Structure Design		Density (kN/m^3^)	Cohesion (kPa)	Internal friction angle (^°^)	Permeability coefficient (m/d)
Sliding mass	Prototype sliding mass	19	21	26	0.25
	Preparing sliding mass	19.1	0.3	25.8	0.03
	Formulation plan	Sandy soil: Clay: Water = 6:3:1			
Sliding belt	Prototype sliding belt	18.5	19	17	//
	Preparing sliding belt	18.7	0.27	16.9	//
	Formulation plan	Barium sulfate: talcum powder: water = 2:4:4			
Sliding bed	Formulation plan	Brick bedrock (only for support, similar materials are not considered)			

### Flume model production and monitoring program

The flume model experiment apparatus comprises a model box, a data monitoring and acquisition system, a rainfall simulation system, and model monitoring system designed to replicate landslide dynamics. Based on the slope structure characteristics of the prototype, the sliding bed was constructed using masonry and finished with cement mortar to ensure a smooth surface (Fig 5a). Subsequently, the sliding belt material was uniformly applied over the sliding bed (Fig 5b). Following the geological cross-section of the main sliding surface, the sliding mass thickness was scaled proportionally, with the soil screened to ensure uniformity (Fig 5c) and thoroughly mixed (Fig 5d) before being layered and compacted (Fig 5e). The slope was then shaped to the predetermined slope morphology (Fig 5f), and sensors, including pull wire displacement sensors and pore water pressure sensors, were installed at designated positions to complete the construction of the landslide model.

**Fig 6:**
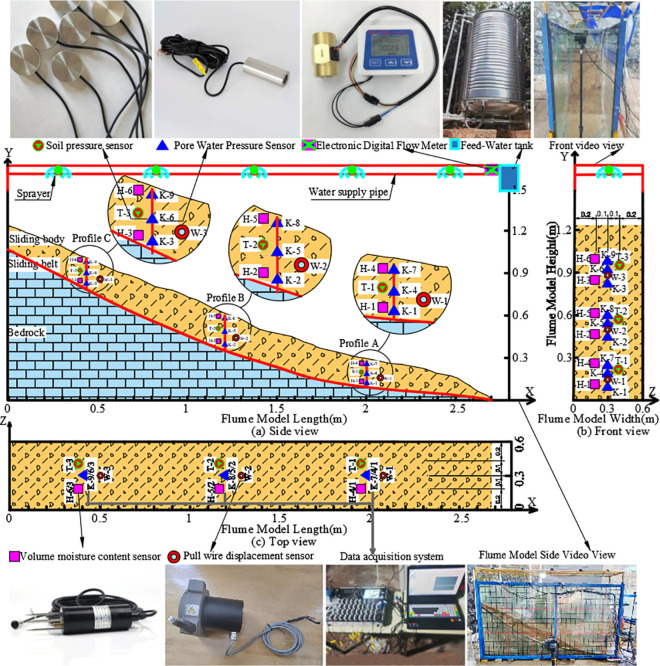
Equipment and instrument layout for the Flume Model test.

To investigate the variations in soil and hydraulic properties across different segments of the landslide under various rainfall patterns, as well as the processes of rainfall infiltration and slope instability, an experimental setup was designed with reference to the automated monitoring points of the Wangjiapo landslide. This ensured that the key profiles of the experimental model aligned with the primary profile and automated monitoring configuration of the actual landslide. Specifically, three vertical monitoring sections (A, B, and C) were established from the front edge to the trailing edge of the slope. Each section was equipped with a suite of sensors, comprising one pull wire displacement sensor, one soil pressure sensor, three pore water pressure sensors, and two volume moisture content sensors. These sensors monitored slope displacement, soil pressure, pore water pressure, and volume moisture content, respectively, validating the appropriateness of the sensor selection.The total thickness of the experimental slope was set at 17 cm. Pore water pressure sensors were positioned at depths of 1 cm, 9 cm, and 16 cm in each monitoring section, while volume moisture content sensors were placed at depths of 2 cm and 15 cm. Soil pressure sensors and pull wire displacement sensors were installed at a depth of 9 cm in each section (Fig 6) for a detailed schematic of the sensor arrangement). This multi-depth sensor configuration enabled the capture of rainwater infiltration effects across various soil layers, encompassing the shallow surface layer, middle layer, and critical zones proximate to the sliding belt. The spatial distribution of sensors at the front edge, central edge, and trailing edge corresponded to the compression zone, main movement zone, and tension zone of the landslide, respectively. This arrangement facilitated a comprehensive characterization of the landslide’s spatial deformation characteristics, thereby demonstrating the rationality and efficacy of the sensor deployment strategy.

A rainfall simulation system was installed above the flume model, comprising an electronic digital flow meter, a water supply pipe, and a sprayer. Model parameters were monitored and recorded in real time using an integrated data acquisition and monitoring system, with technical specifications detailed in Table 3. To ensure data reliability, all sensors were calibrated in the laboratory and installed in strict accordance with standardized protocols. These protocols included sensor saturation treatment, minimizing gaps between the soil and sensors, and positioning sensors perpendicular to the primary sliding direction of the model to reduce systematic errors and enhance measurement accuracy. Given the model slope’s dimensions—17 cm in thickness and 2.7 m in length—the anticipated displacement and pressure variations are minimal. High-precision sensors were employed to effectively capture these subtle changes, further ensuring measurement accuracy.The three monitoring profiles, spanning the front edge, central edge, and trailing edge, combined with multi-depth sensor placements, comprehensively characterize the spatial and vertical deformation characteristics of the landslide. The current sensor coverage and density under laboratory conditions are sufficient to meet experimental requirements. This sensor deployment strategy provides robust data support for investigating the deformation mechanisms of seepage-induced landslides.

**Table 3:** Technical parameters of flume model experimental instrument.

System Unit	Instruments	Model	Number	Key Technical Parameters
Rainfall simulation system	Electronic Digital Flow Meter	HHKM	1	operating voltage range: 1.8V–3.5V, flow range: 1–30L/min, accuracy: ≤ 5–10%
	Water supply pipe	SL	5	Diameter: 4mm; Length: 20 meters
	Sprayer	TW3010	5	Diameter: 0.3 mm; rainfall intensity: 0–10 mm/h
Model monitoring system	Camera	SONY-ILCE-6000	2	APS frame: 23.5 × 15.6 mm; maximum resolution: 6000 × 4000; optical zoom: 1–16 times
Data monitoring and acquisition system	Soil Pressure Sensor	DMTY	3	output voltage: 0–5 V; range: 0–50 kPa; accuracy: ≤ 0.5% F.S
	Pore Water Pressure Sensor	DMKY	9	output voltage: 0–5 V; range: 0–10 kPa; accuracy: ≤ 0.1% F.S
	Volume moisture content sensor	DM-SA01	6	output voltage: 0–5 V; range: 0–100%; accuracy: ≤ 2%
	Pull wire Displacement sensor	P4SHT4	3	pull-out speed: 1500 mm/s; accuracy: ≤ 0.05%
	Data acquisition system	DM-YB1840	1	voltage: 2V; range: 1–19999*μ*; accuracy: ≤ 0.05% F.S
Model box	Model box	PPMXX	1	Length, width, and height: 2.8m, 0.6m, 1.5m

### Test rain pattern design

In this study, rainfall monitoring data from Xingshan County in 2017 (Fig 4) were analyzed to characterize distinct rainfall patterns. The rainfall parameters for key dates are presented in Table 4.

**Table 4:** Rainfall parameters under key date nodes.

Date(year/month/day)	Rainfall duration (h)	Rainfall amount (mm)	Actual rainfall intensity (mm/h)	Design rainfall intensity (mm/h)
2017/8/7	0.68	46	46/0.68 ≈ 67.6	67.6/$\sqrt{70}$ ≈ 8
2017/8/8	0.49	20	20/0.49 ≈ 40.8	40.8/$\sqrt{70}$ ≈ 4.9
2017/8/9	0.48	8	8/0.48 ≈ 16.7	16.7/$\sqrt{70}$ ≈ 2
2017/9/4	0.32	13	13/0.32 ≈ 40.6	40.6/$\sqrt{70}$ ≈ 4.9
2017/9/17	0.45	7.8	7.8/0.45 ≈ 17.3	17.3/$\sqrt{70}$ ≈ 2.1
2017/9/18	0.35	15.2	15.2/0.35 ≈ 43.4	43.4/$\sqrt{70}$ ≈ 5.2
2017/10/2	0.48	8	8/0.48 ≈ 16.7	16.7/$\sqrt{70}$ ≈ 2
2017/10/4	0.5	21	21/0.5 ≈ 42	42/$\sqrt{70}$ ≈ 5
2017/10/5	0.43	29	29/0.43 ≈ 67.4	67.4/$\sqrt{70}$ ≈ 8.1

These rainfall patterns were derived from the precipitation characteristics observed on specific dates. Using the similarity theory, the actual rainfall intensity was scaled to the model dimensions with a similarity ratio of $1/\sqrt{70}$ to determine the design rainfall intensity. Various design rainfall intensities were simulated using an electronic digital flow meter and a sprayer. This approach ensured the accuracy of the hydraulic and mechanical responses in the flume model, effectively replicating the rainfall conditions observed in the Three Gorges Reservoir area. The design rainfall pattern conditions and their corresponding durations are detailed in Table 5.

**Table 5:** Rainfall pattern conditions setup.

Rain Pattern Number	Rainfall Duration (min)	Design Rainfall Intensity (mm/h)	Rainfall Pattern
Rainfall pattern I	0 → 90	5	Uniform rainfall
Rainfall pattern II	0 → 45	8 → 5	Decreasing rainfall
	45 → 90	5 → 2	
Rainfall pattern III	0 → 45	2 → 5	Incremental rainfall
	45 → 90	5 → 8	
Rainfall pattern IV	0 → 45	2 → 5	Single-peak rainfall
	45 → 70	5	
	70 → 115	5 → 2	

## Model test results

### Macroscopic deformation process of landslide

**Fig 7:**
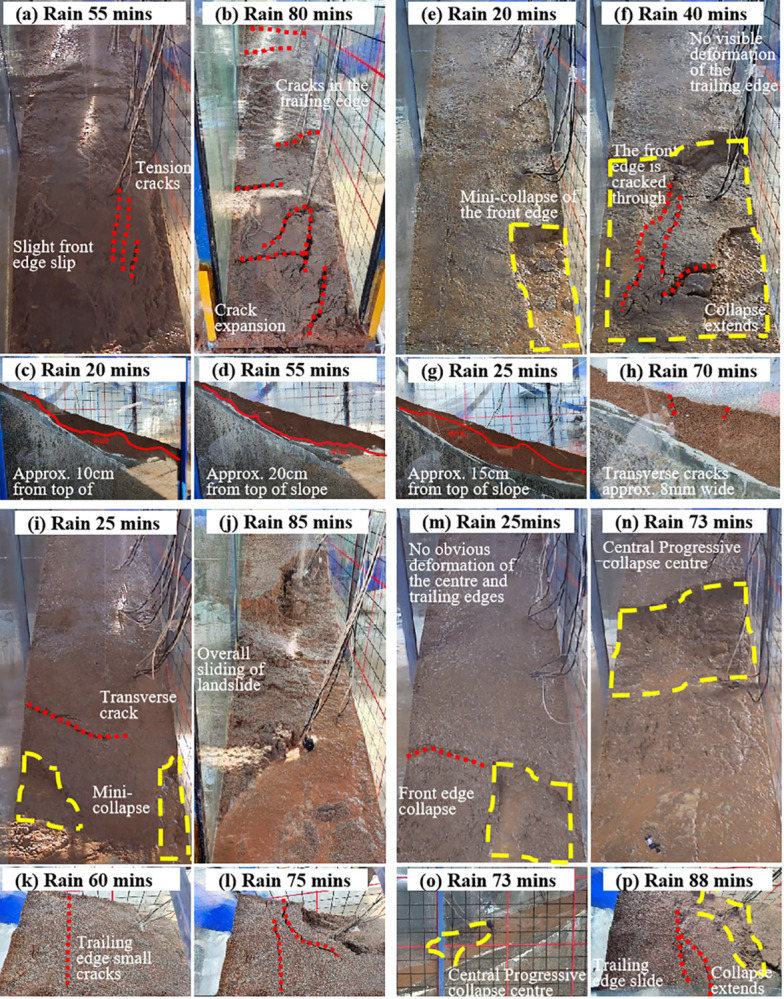
Macroscopic deformation process of landslide under different rain patterns: (a-d) Uniform rainfall, (e-h) decreasing rainfall, (i-l) incremental rainfall, (m-p) single-peak rainfall.

Under uniform rainfall, after 20mins, the infiltration line was approximately 10 cm from the top of the slope, accompanied by erosion and minor tensile cracks at the front edge of the landslide (Fig 7c). By 55mins, fine grooves and multiple tensile cracks developed on the left side of the slope surface (Fig 7a), with the front edge exhibiting slight displacement and the infiltration line reaching approximately 20 cm from the top of the slope (Fig 7d). At 71mins, the fine grooves on the left side deepened and widened, and the saturation zone extended to the deposit-bedding interface. By 80mins, tensile cracks emerged at the trailing edge, while new cracks continued to form on the slope surface. Existing cracks expanded into transverse shear tension cracks, and soil at the slope foot began to slide, ultimately leading to the instability and collapse of the entire slope (Fig 7b).

Under decreasing rainfall, after 20mins, a mini-collapse occurred at the front edge of the landslide on the left side (Fig 7e). By 25mins, the infiltration line was approximately 15 cm from the slope surface (Fig 7g). As rainfall intensity diminished, by 40mins, rainfall infiltration and surface runoff stabilized, resulting in continuous cracks at the front edge. This caused sliding at the slope foot, triggering a collapse of the entire front edge (Fig 7f), with no significant deformation observed at the trailing edge. At 70mins, transverse shear tension cracks appeared at the trailing edge (Fig 7h). By 80mins, the landslide became unstable and failed. Although the onset of failure was earlier under decreasing rainfall, the overall failure duration was comparable to that under uniform rainfall.

Under incremental rainfall, after 25mins, mini-collapses accompanied by transverse shear tension cracks developed on both sides of the front edge (Fig 7i). As rainfall intensity increased, the extent of collapse at the slope foot expanded progressively. By 60mins, fine transverse cracks, approximately 2 mm wide, emerged at the trailing edge (Fig 7k). At this stage, the soil mass at the front edge was compromised, forming a free-standing surface that collapsed further under rainwater action. By 75mins, the cracks at the trailing edge interconnected and widened to approximately 5 mm (Fig 7l). At 85mins, the entire landslide failed due to instability (Fig 7j). Owing to the lower initial rainfall intensity, the overall failure time under incremental rainfall was approximately 5mins later than that under uniform and decreasing rainfall patterns.

Under single-peak rainfall, after 25mins, a mini-collapse occurred at the front edge of the slope, with no significant deformation observed at the central or trailing edges (Fig 7m). By 65mins, small tensile cracks developed at the trailing edge and extended progressively. At 73mins, progressive collapse initiated at the central edge (Figs 7n and 7o). By 88mins, the trailing edge began to slide (Fig 7p). At 105mins, the entire landslide became unstable and failed. Compared to incremental rainfall, the overall failure time under single-peak rainfall was delayed by approximately 20mins.

### Displacement characteristics of the slope body

**Fig 8:**
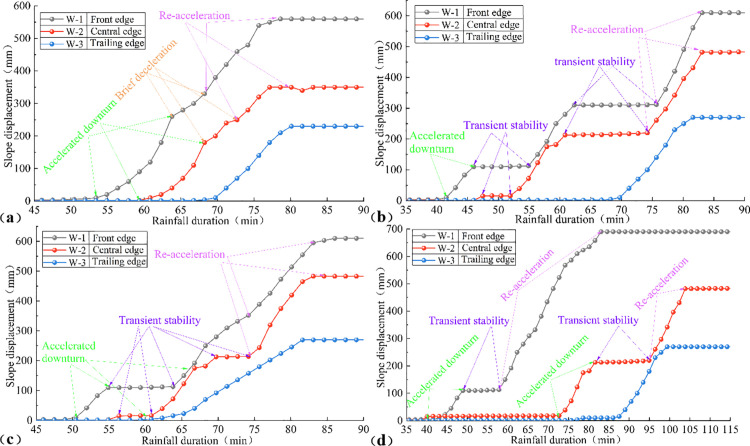
Time-varying curves of slope displacement under different rainfall patterns: (a) Uniform rainfall, (b) decreasing rainfall, (c) incremental rainfall, (d) single-peak rainfall.

Analysis of macro-scale deformation characteristics reveals spatial variability in slope displacement during the failure process. To capture this variability, pull wire displacement sensors (W-1, W-2, and W-3) were strategically deployed at the front edge, central edge, and trailing edge of the landslide, respectively, as observation points. These sensors facilitated the generation of a comprehensive slope displacement monitoring curve, presented in Fig 8.

Under varying rain patterns, the displacement sequence of the landslide model progressed from the front edge to the central edge, and finally to the trailing edge. Time-displacement curves for the trailing edge exhibited consistent patterns across all rain patterns, characterized by an initial stable phase indicative of uniform deformation, followed by a gradual flattening phase representing failure arrest. Under uniform rainfall, the failure process at the front and central edges followed a sequence of accelerated downturn, brief deceleration, re-acceleration, and ultimate stabilization (Fig 8a). In contrast, under other rain patterns, the displacement curves for the front and central edges displayed a transient stability segment. This stability resulted from soil accumulation at the slope foot, where the resistance force of the soil in the front and central edges exceeded the sliding force, temporarily impeding displacement (Figs 8b to 8d).

### Variation characteristics of volume moisture content

**Fig 9:**
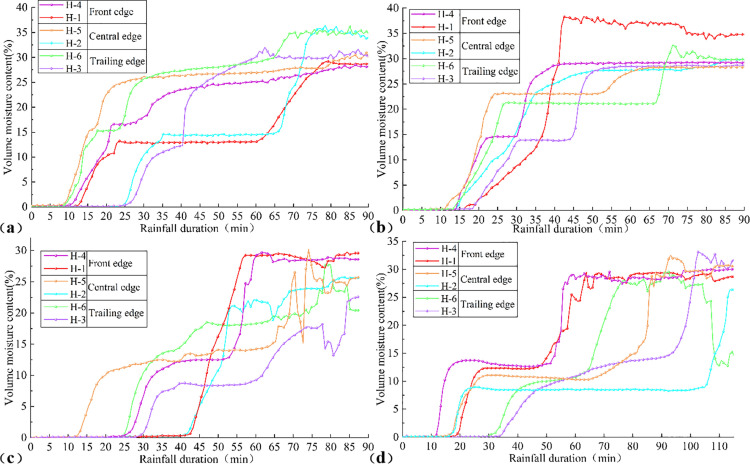
Time-varying curves of volumetric moisture content under different rainfall patterns: (a) Uniform rainfall, (b) decreasing rainfall, (c) incremental rainfall, (d) single-peak rainfall.

Variations in volume moisture content influence the cohesion and internal friction angle of the soil, thereby disrupting the stress-strain equilibrium of the slope. Volume moisture content sensors (H-1/H-4, H-2/H-5, H-3/H-6) were installed at the front edge, central edge, and trailing edge of the slope, respectively, to monitor these changes. Temporal variations in volume moisture content under different rainfall patterns are illustrated in Fig 9.

Under uniform rainfall (Fig 9a), after 8.5mins, the volume moisture content at the surface of the trailing edge began to increase. By 11mins, the surface layer at the front edge exhibited a response in volume moisture content. At 22mins, the volume moisture content curves for the slope surface stabilized, while the moisture content at deeper levels of the slope rose rapidly. By 80mins, fluctuations in volume moisture content across various slope locations culminated in overall instability and failure of the landslide.

Under decreasing rainfall (Fig 9b), after 20mins, the volume moisture content at the surface of the front edge remained stable, whereas the moisture content at deeper levels continued to rise. This pattern resulted from a mini-collapse at the front edge, which facilitated downward seepage of moisture to the sliding belt, approaching saturation, while the surface layer achieved equilibrium between seepage and rainfall replenishment. At 70mins, the expansion of transverse shear tension cracks at the trailing edge provided enhanced seepage pathways, causing the volume moisture content at the surface of the trailing edge to transition from stable to rapidly increasing.

Under incremental rainfall (Fig 9c), between 42.5 and 61mins, the volume moisture content at the central edge and front edge increased rapidly, driving the landslide into an accelerated failure stage due to the formation of a free-standing surface.

Under single-peak rainfall (Fig 9d), the volume moisture content curves across different parts of the landslide exhibited consistent trends, characterized by an initial increase, stabilization, a subsequent rise, and final fluctuation followed by stabilization.

Analysis of Fig 9 reveals that, across all rain patterns, the front edge is more susceptible to rainfall infiltration than the trailing edge, with surface-level volume moisture content responding earlier than at deeper levels.

### Variation characteristics of slope stress

Pore water pressure sensors (K-1/K-4/K-7, K-2/K-5/K-8, K-3/K-6/K-9) were deployed at monitoring profiles A, B, and C, respectively, to measure pore water pressure dynamics. Similarly, soil pressure sensors (T-1, T-2, T-3) were installed at profiles A, B, and C, respectively, to assess soil pressure variations.Prior to the experiment, the initial readings of all sensors were reset to zero to establish a baseline. Consequently, the monitoring data primarily serve to analyze trends in stress variations within the slope rather than absolute stress magnitudes [57]. The data collected under various rain patterns are analyzed below.

#### Pore water pressure.

**Fig 10:**
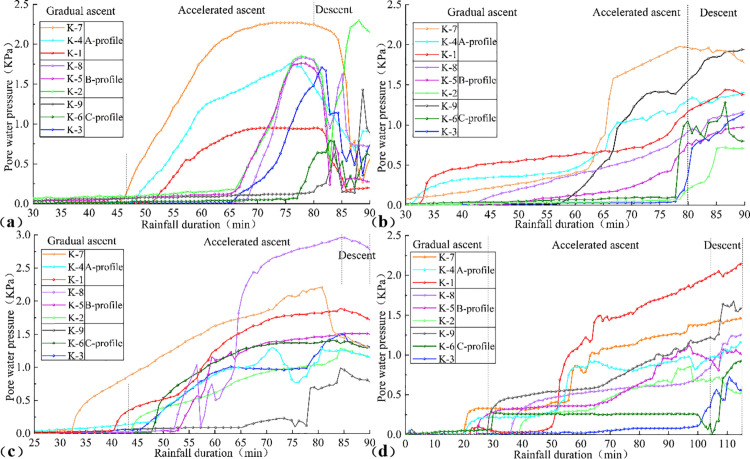
Time-varying curves of pore water pressure under different rainfall patterns: (a) Uniform rainfall, (b) decreasing rainfall, (c) incremental rainfall,(d) single-peak rainfall.

The temporal variation of pore water pressure under different rain patterns is illustrated in Fig 10, elucidating the impact of rainfall infiltration on the hydraulic properties of the slope. Analysis of the curves indicates that the evolution of pore water pressure within the slope can be categorized into three distinct phases: gradual increase, accelerated increase, and decline. During the initial stage of rainfall, residual air within the slope hinders the formation of effective seepage pathways, causing rainwater to infiltrate primarily through tensile cracks on the slope surface. This leads to a gradual increase in pore water pressure (u), which slowly reduces the effective stress ($\sigma^{\prime }=\sigma -u$). Per the Mohr-Coulomb criterion ($\tau =c^{\prime }\;+\;\sigma^{\prime }\tan\phi$) [58,59], this reduction progressively diminishes the shear strength (*τ*) of the soil. As rainfall persists, seepage pathways develop, allowing rainwater to infiltrate along landslide cracks to the sliding surface, elevating the groundwater level. Consequently, pore water pressure (u) increases rapidly, causing a sharp decline in effective stress ($\sigma^{\prime }$) and further degradation of the soil’s shear strength. In the later stages of rainfall, the landslide undergoes complete failure, enabling rapid dissipation of pore water. The pore water pressure curve peaks and subsequently declines, with the effective stress ($\sigma^{\prime }$) potentially reaching a critical threshold prior to the decline in pore water pressure (u), ultimately resulting in the loss of soil strength.

Although the pore water pressure variation curves across different locations exhibited similar trends, their response times varied significantly. The pore water pressure response at the front edge and central edge of the landslide occurred markedly earlier than at the trailing edge, with surface-level responses preceding those at deeper levels. This temporal disparity arises because rainfall infiltration occurs not only vertically through the slope surface but also horizontally from the trailing edge toward the front edge. Consequently, pore water accumulates more rapidly at the front edge, leading to earlier saturation compared to other regions.

#### Soil pressure.

**Fig 11:**
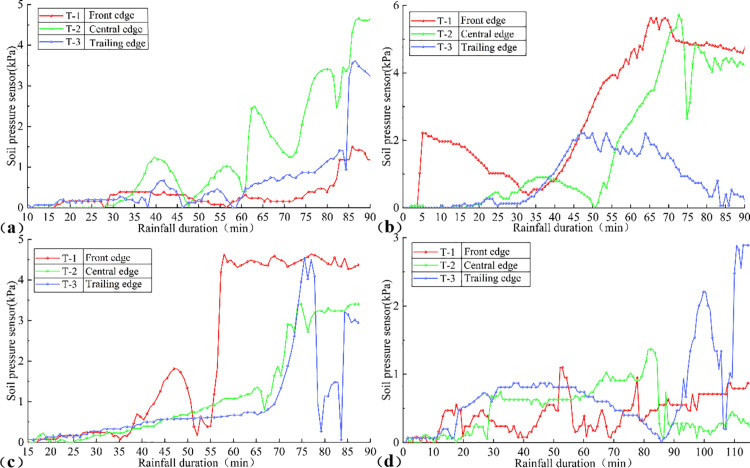
Time-varying curves of Soil pressure under different rainfall patterns: (a) Uniform rainfall, (b) decreasing rainfall, (c) incremental rainfall, (d) single-peak rainfall.

The temporal evolution of soil pressure under various rain patterns is illustrated in Fig 11, reflecting the effect of rainwater infiltration on the soil properties of the slope.

Under uniform rainfall (Fig 11a), effective seepage pathways had not yet formed during the initial stage, resulting in slow rainfall infiltration and incomplete soil saturation. Consequently, pore water pressure (u) increased gradually, while soil pressure (*σ*), primarily governed by the self-weight of the soil, rose slowly. As rainfall persisted, tensile cracks within the slope expanded, leading to a rapid increase in pore water pressure (u) that reached a peak. The soil transitioned from an unsaturated to a saturated state, increasing its weight and causing a continuous rise in soil pressure (*σ*). However, the increase in (*σ*) was less pronounced than that of (u), resulting in a decline in effective stress ($\sigma^{\prime }$). This strength degradation triggered the complete failure of the landslide at 80mins.

Under decreasing rainfall (Fig 11b), soil pressure (*σ*) at the front edge increased rapidly in the initial stage due to enhanced soil weight from rainfall infiltration, which also elevated pore water pressure (u). Subsequently, (*σ*) decreased gradually as mini-collapses and deformation on the left side of the front edge induced stress relaxation and stabilization. These collapses formed new drainage pathways, causing a slower rise in the pore water pressure (u) curve. The pattern of rapid (*σ*) increase followed by a gradual decrease reflects stress release due to front-edge collapse, with the rise in (u) reducing effective stress ($\sigma^{\prime }$) and degrading soil strength, leading to localized instability. As the sliding mass compacted the soil at the front edge, soil pressure (*σ*) rose again, potentially partially restoring ($\sigma^{\prime }$). However, repeated cycles of soil loosening and compaction continued to degrade soil strength until failure ceased.

Under incremental rainfall (Fig 11c), initial rainfall infiltration was slow, resulting in a slight increase in soil weight, low pore water pressure (u), and a gradual rise in soil pressure (*σ*). As rainfall intensity increased, increased rainwater infiltration caused a rapid rise in pore water pressure (u) and a sharp increase in soil pressure (*σ*).With sustained rainfall, the escalating pore water pressure (u) reduces effective stress ($\sigma^{\prime }$), and the progressive extension and interconnection of tension cracks further diminish local soil strength. This degradation of soil strength propagates, resulting in fluctuations in the soil pressure curve. Concurrently, effective stress ($\sigma^{\prime }$) fluctuates due to drainage and pressure variations, This pattern indicates a gradual failure process, underscoring the stress redistribution within the slope.

Under single-peak rainfall (Fig 11d), initial rainfall infiltrated through tensile cracks on the slope surface, increasing pore water pressure (u) and causing a gradual rise in soil pressure (*σ*). As rainfall continued, cracks extended and interconnected, triggering progressive collapse at the front edge and central edge, forming a free-standing surface. This collapse induced further progressive failure of the trailing edge soil. Each collapse event caused a sudden decrease in soil pressure (*σ*), followed by rearrangement and compaction of the remaining soil, leading to a subsequent rise in (*σ*). These repeated cycles resulted in multiple abrupt fluctuations in the soil pressure curve, corresponding to layer-by-layer progressive collapse. Each collapse reduced effective stress ($\sigma^{\prime }$), degrading soil strength and reflecting a progressive failure pattern.

### Statistical processing of experimental data

This study employed a flume model to simulate landslide deformation processes under four distinct rainfall patterns, with monitoring data acquired from multiple sensors positioned at various depths across the front edge, central edge, and trailing edge of the landslide. Key metrics, including initial response time, deformation response rate,lag time of deformation response, and pore water pressure,, were extracted from the experimental monitoring data to systematically assess the differential impacts of rainfall patterns on landslide deformation. Statistical summaries of these critical metrics for each rainfall pattern are presented in Table 6.

**Table 6:** Statistics of key indicators of tests under different rain patterns.

Key indicators	Initiation Response Time of Deformation at the Leading Edge/Middle/Trailing Edge (min)	Deformation Response Rate at the Leading Edge/Middle/Trailing Edge (mm/min)	Lag Time of Deformation Response in the Lower Part of the Leading/Middle/Trailing Edge Relative to the Upper Part (min)	Lag Time of Deformation Response at the Trailing Edge Relative to the Leading Edge (min)
**Uniform rainfall**	47.4/59.4/66.7	17.9/17/17.2	2.9/14.7/15.4	19.3
**Decreasing rainfall**	40/46/68	26.6/22.1/20	4.4/4.4/5.9	28.0
**Incremental rainfall**	48.9/55/60.8	22.4/22.1/13	17.5/29.4/4.4	11.9
**Single-peak rainfall**	41.5/72.7/77	20.5/22/18	5.8/1.4/4.4	35.5
**Key indicators**	**Total Instability Time (min)**	**Initiation Response Time of Pore Water Pressure in the Upper Part of the Leading Edge/Middle/Trailing Edge (min)**	**Initiation Response Time of Pore Water Pressure in the Lower Part of the Leading Edge/Middle/Trailing Edge (min)**	**Time Period of Accelerated Pore Water Pressure Increase (min)**
**Uniform rainfall**	80	45.5/63.2/65.8	51.3/66/65.8	46.5 → 80
**Decreasing rainfall**	80	30.8/42.5/57	32.3/66.7/77.7	61.5 → 80
**Incremental rainfall**	85	32.2/39.6/44	40.3/44/45.5	44.2 → 85
**Single-peak rainfall**	105	19.8/35.9/28.7	22/38.9/74	28 → 104

#### Deformation response time.

The initiation of deformation responses in landslides under various rainfall patterns exhibits a consistent spatial progression from the front edge to the central edge and trailing edge, as well as from the upper layer to the lower layer. Across four rainfall patterns, the lag in deformation response time at the trailing edge relative to the front edge is 19.3, 28.0, 11.9, and 35.5mins, respectively. Furthermore, the lag in deformation response time for the lower layer compared to the upper layer at the front edge, central edge, and trailing edge is as follows: 2.9/14.7/15.4mins, 4.4/4.4/5.9mins, 17.5/29.4/4.4mins, and 5.8/1.4/4.4mins, respectively. The deformation and propagation patterns of landslides remained consistent across all four rainfall patterns, indicating a progressive influence of rainfall infiltration on landslide instability. The results suggest that rainfall initially impacts the upper layer and front edge of the slope.

#### Deformation response rate and overall instability time.

Regarding the deformation response rate and overall instability time, landslides under uniform rainfall exhibited similar deformation response rates across different sections, with a shorter overall instability time compared to other rainfall patterns. For instance, under uniform rainfall, the overall instability time was 80 minutes, with deformation response rates at the front edge, central edge, and trailing edge of 17.9, 17.0, and 17.2 mm/min, respectively. These findings indicate that uniform rainfall induces relatively rapid overall instability, characterized by smooth sliding across the landslide. In contrast, other rainfall patterns significantly influenced the deformation process. From decreasing rainfall to incremental rainfall to single-peak rainfall, the overall instability time progressively increased, while the deformation response rates at various sections gradually decreased, exhibiting a declining trend from the front edge to the trailing edge. Specifically, under decreasing rainfall, the overall instability time was 80 minutes, with deformation response rates of 26.6, 22.1, and 20.0 mm/min at the front edge, central edge, and trailing edge, respectively. Under incremental rainfall, the overall instability time was 85 minutes, with rates of 22.4, 22.1, and 13.0 mm/min, respectively. For single-peak rainfall, the overall instability time extended to 105 minutes, with rates of 20.5, 22.0, and 18.0 mm/min, respectively. These results suggest that landslide deformation follows a progressive collapse process, initiating at the front edge and extending to the trailing edge, with non-uniform rainfall patterns introducing varying degrees of delay in overall landslide instability.

#### Pore water pressure response time and acceleration period.

Analysis of pore water pressure response times and acceleration phases revealed a consistent propagation trend across different rainfall patterns, with initial pore water pressure responses spreading from the front edge to the central edge and then to the trailing edge, and from the upper layer to the lower layer. This pattern reflects the stress changes induced by rainfall infiltration into the landslide. For example, under incremental rainfall, the pore water pressure response times for the upper and lower layers at the front edge, central edge, and trailing edge were 32.2, 39.6, and 44.0 minutes, and 40.3, 44.0, and 45.5 minutes, respectively. The acceleration phase of pore water pressure varied across rainfall patterns. During this phase, tensile cracks on the slope surface progressively increased and interconnected, accelerating the landslide’s downhill movement until complete instability was reached. This indicates that a rapid increase in pore water pressure is detrimental to landslide stability. For instance, under uniform rainfall, the rapid rise in pore water pressure resulted in a shorter overall instability time, whereas under single-peak rainfall, the slower increase in pore water pressure led to a longer instability time and a more gradual failure process.

### Comparison of test results with actual monitoring data

To validate the model test results and gain a deeper understanding of the deformation mechanism of the Wangjiapo landslide, this section compares the model test data with in-situ monitoring data collected from July to November 2017. The in-situ data were obtained from the Yichang Geological Disaster Professional Monitoring and Early Warning System, ensuring a robust dataset for assessing the model’s accuracy and enhancing understanding of the landslide’s deformation behavior.

#### Landslide deformation characteristics and mechanisms.

Flume model experiments demonstrate that uniform rainfall induces smoother sliding of landslides, whereas other rainfall patterns trigger initial failure at the front edge, leading to progressive collapse toward the trailing edge. Field monitoring data from the Wangjiapo landslide (Fig 4), specifically from September 4–6, 2017, reveal that under uniform rainfall, the cumulative displacement at monitoring points W1-1 and W2 increased steadily by 54 mm and 43 mm, respectively, over three days. Compared to other periods (August 7–9, September 17–19, and October 2–5, 2017), the displacement curves indicate that W1-1 exhibited earlier displacement than W2, and W2 preceded W3, consistent with the deformation characteristics observed under different rainfall patterns in the model experiments. Furthermore, the front edge monitoring points (W1-1 and W2) exhibited significantly larger deformations, with cumulative displacements of 1067.7 mm and 2370 mm, respectively, compared to 369.2 mm at the trailing edge (W3). These findings indicate greater deformation at the front of the landslide than at the trailing edge, reflecting a retrogressive landslide failure pattern, which aligns with the spatial characteristics of landslide instability observed in the model experiments.

#### Landslide instability factors

Model experiments further reveal that rainfall infiltration elevates pore water pressure, reduces shear strength, and ultimately induces slope instability. Field monitoring data from the Wangjiapo landslide (Fig 4) corroborate that rainfall infiltration increases pore water pressure, diminishes effective stress in the soil, significantly weakens shear strength, and accelerates landslide displacement. The cumulative displacement curves for monitoring points W1-1, W2, and W3 exhibit step-like displacement characteristics. For instance, the cumulative displacement curve for W2 displayed four distinct steps of varying magnitude between July 8–28, August 7–18, September 9–15, and September 24–October 22, 2017. These patterns are consistent with the model experiments, where increased pore water pressure was identified as a key inducing factor for landslide instability.

### Numerical simulation validation

**Fig 12:**
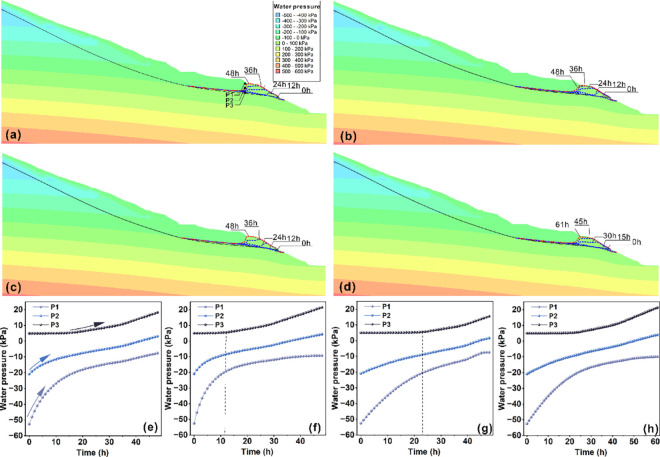
Numerical simulation results of the seepage flow field for the Wangjiapo landslide under different rain patterns: (a)–(d) illustrate the pore water pressure distribution under uniform rainfall, decreasing rainfall, incremental rainfall, and single-peak rainfall, respectively; (e)–(h) present the pore water pressure time-history curves corresponding to the four rain patterns.

In this study, the SEEP/W module of the finite element-based Geo-Studio software was utilized to conduct a numerical simulation of the dynamic evolution of the seepage field in the Wangjiapo landslide under four distinct rain patterns: uniform rainfall, incremental rainfall, decreasing rainfall, and single-peak rainfall. The simulation results reveal that rainfall infiltration initially induces a response at the front edge of the landslide, with the transient saturated zone progressively extending toward the trailing edge and deeper layers, as illustrated in Fig 12a– 12d. The groundwater level exhibits a continuous rise over time. Furthermore, analysis of the pore water pressure time-history curves (Fig 12e– 12h) for monitoring points P1 (shallow layer), P2 (middle layer), and P3 (deep layer, near the sliding belt) within the same profile demonstrates a depth-dependent lag effect in pore water pressure response. Specifically, the response is most rapid in the landslide’s surface layer and diminishes with increasing depth, consistent with the findings from the flume model experiment. Comparative analysis of the different rain patterns reveals significant variations in the seepage field and pore water pressure response characteristics. Notably, under incremental rainfall conditions, the initially low rainfall intensity results in slower water infiltration, leading to a pronounced lag in the rise of the groundwater level and the increase in pore water pressure near the sliding belt.

In summary, rainfall infiltration, which elevates pore water pressure within the landslide, emerges as a crucial factor driving landslide deformation and instability. Rainfall intensity and rainfall time series directly influence the pore water pressure response patterns. Infiltration diminishes or eliminates matrix suction in the shallow unsaturated zone, while simultaneously raising the groundwater level and increasing positive pore water pressure in the saturated zone. The combined effect of these processes reduces the effective stress in the rock and soil mass, significantly diminishing shear strength and ultimately inducing landslide deformation and failure.

## Discussion

### Discussion of the deformation characteristics of landslides

Rainfall is a crucial factor in triggering accumulated landslides and significantly influences the deformation and stability of landslide bodies. This study reveals pronounced differences in the effects of various rainfall patterns on landslide deformation. The deformation process of accumulated landslides typically manifests as a stepwise instability pattern, progressing from the surface to deeper layers and from local to overall instability. Under different rainfall patterns, this process exhibits evolutionary characteristics, propagating from the front edge to the trailing edge and from the upper layer to the lower layer, consistent with the mechanical response characteristics of landslides.

Under uniform rainfall, rainwater infiltrates evenly, and the hydraulic response of the landslide body is relatively consistent. Cracks form first at the front edge and gradually expand. As rainfall continues, the front edge of the landslide gradually develops from the front edge to the rear edge due to the decrease in effective stress and the weakening of shear strength, and eventually slides along the sliding mass base covering surface. This is similar to the conclusions of previous studies [57] on the effect of rainfall on landslide stability.Under decreasing rainfall, rapid infiltration saturates the front-edge soil, inducing tension cracks and localized mini-collapse. The trailing edge exhibits minimal deformation initially, but as rainfall intensity diminishes, moisture infiltration triggers progressive instability, manifesting as a gradual failure process. Conversely, under incremental rainfall, tension cracks at the front edge develop and accelerate, with increasing soil saturation and decreasing shear strength driving progressive destabilization from the front edge to the trailing edge. Although slower than the instability process under decreasing rainfall, this process escalates rapidly following an initial collapse at the front edge. Under single-peak rainfall, early minor rainfall induces mini-collapse at the front edge of the landslide. As rainfall intensity peaks, tension cracks propagate inward, destabilizing the soil and forming a sliding surface, culminating in overall failure. This failure mode resembles that under incremental rainfall, but variations in rainfall intensity delay the onset of complete instability.

### Discussion of the failure mechanism of the landslide

Rainfall intensity and duration significantly govern the instability processes of landslides, exhibiting distinct phased characteristics. Initially, rainfall induces localized hydraulic failure at the front edge, which intensifies as infiltration persists. Progressive water accumulation in the surface soil elevates saturation to a critical threshold, rapidly reducing shear strength and triggering overall structural instability. Different rainfall patterns elicit distinct deformation characteristics and instability processes in landslides.

Under the uniform rainfall, instability primarily results from elevated pore water pressure at the bedrock surface. Continuous infiltration reduces effective stress within the sliding mass, diminishing soil strength. As pore water pressure accumulates, shear strength declines, forming a sliding surface [60] and culminating in overall instability. This failure mode manifests as deformation at the front edge, triggering sliding of the entire landslide body, consistent with the response patterns noted in previous studies [57], though distinct in its failure mechanism. Conversely, under decremental rainfall, incremental rainfall, and single-peak rainfall, prolonged infiltration saturates the surface soil, reducing shear strength and initiating hydraulic damage at the front edge. This triggers progressive collapse toward the trailing edge, driven by the combined effects of hydraulic action and soil instability [61]. The duration and intensity of different rainfall patterns affect the failure sequence of landslides, causing the rear part of the landslide to begin to deform significantly after the front edge has failed. Under these three rain patterns, the failure mode of the landslide was characterized by the front edge failing first, followed by the gradual collapse of the rear edge.

### Research limitations and future research directions

Although this study revealed the deformation mechanism of accumulated landslides under different rainfall patterns through a flume model test system, filling a gap in the field of complex rainfall pattern research, there are still some limitations.First, due to the limitations of the experimental scale and simplified boundary conditions, the Flume Model experiments could not fully simulate the complex geological factors of actual landslides, such as soil heterogeneity, micro-landform features, and the slope-stabilizing effect of vegetation roots. These factors may significantly affect the rainfall infiltration rate, pore water pressure distribution, and landslide stability.Second, the study focused on four typical rainfall patterns: uniform, decreasing, incremental, and single-peak. However, rainfall patterns in nature are more complex, such as multi-peak rainfall, intermittent rainfall, or a combination of extreme rainfall and low-intensity rainfall, whose spatiotemporal distribution may have a more significant impact on landslide deformation and instability, which needs further study.In the future, flume model experiments should be optimized to more realistically reproduce complex geological factors, expand research on more complex rainfall patterns, further improve the universality and application value of the results, and provide a more accurate scientific basis for landslide disaster prediction and prevention.

## Conclusions

This study innovatively employed a flume model to simulate varied rainfall patterns, systematically elucidating the deformation mechanisms of accumulated landslides under different temporal rainfall distributions. By addressing a critical gap in existing research that had focused primarily on rainfall intensity rather than rainfall patterns, these findings offer novel insights into landslide behavior under complex hydrological conditions. The following conclusions were drawn:

(1) Landslide failure progresses through four stages—preliminary creep, front-edge collapse, progressive collapse, and final instability [62]. Rainfall intensity and sequence critically influence this process, with the incremental rainfall and the single-peak rainfall promoting a progressive failure pattern.

(2) Rapid increases in pore water pressure are crucial factors in triggering landslide instability [63]. Under the uniform rainfall, elevated pore water pressure reduces the resistance force, prompting overall instability.In contrast, under incremental, decreasing, and single-peak rainfall patterns, initial mini-collapse occurs at the front edge, followed by progressive destabilization as rising pore water pressure and diminishing shear strength exacerbate landslide instability.

(3) Rainfall patterns significantly affect landslide deformation. The uniform rainfall induces relatively stable overall sliding, whereas the decremental rainfall, the incremental rainfall, and the single-peak rainfall trigger initial failure at the front edge, followed by progressive collapse toward the trailing edge.

(4) The instability mechanism of accumulated landslides exhibits complex spatial and temporal dynamics. Spatially, deformation initiates at the front edge and upper layer, with delayed responses at the trailing edge and lower layer. Temporally, overall instability times vary by rainfall pattern: the uniform rainfall and the decremental rainfall result in rapid instability, while the incremental rainfall and the single-peak rainfall exhibit delays of approximately 5 and 25mins, respectively.

(5) This study establishes a methodological framework for landslide monitoring and early warning while demonstrating its practical value in landslide disaster risk assessment and management. Specifically, for engineering control under non-uniform rainfall patterns, such as decreasing, incremental, and single-peak rainfall, we recommend enhancing drainage infrastructure at the front edge to mitigate the cumulative effects of pore water pressure. For early warning and monitoring, under uniform rainfall, a comprehensive surface displacement monitoring network is advised to prioritize the detection of signals indicative of overall slope sliding. For other rainfall patterns, monitoring should focus on early signs of deformation and pore water pressure dynamics. Furthermore, the research findings support the development of regional rainfall threshold models, facilitating the formulation of targeted emergency response strategies and enhancing the timeliness of landslide early warning and risk management.
